# Associations between lifetime classic psychedelic use and markers of physical health

**DOI:** 10.1177/0269881121996863

**Published:** 2021-03-09

**Authors:** Otto Simonsson, James D Sexton, Peter S Hendricks

**Affiliations:** 1 Department of Sociology, University of Oxford, Oxford, UK; 2 Department of Brain Sciences, Imperial College London, London, UK; 3 Department of Health Behavior, University of Alabama at Birmingham, Birmingham, USA

**Keywords:** Classic psychedelics, psilocybin, LSD, health, body mass index, cancer, heart disease

## Abstract

**Background:**

In recent years, there has been significant research on the mental health effects of classic psychedelic use, but there is very little evidence on how classic psychedelics might influence physical health.

**Aims:**

The purpose of the present study was to investigate the associations between lifetime classic psychedelic use and markers of physical health.

**Methods:**

Using data from the National Survey on Drug Use and Health (2015-2018) with 171,766 (unweighted) adults aged 18 or above in the United States, the current study examined the associations between lifetime classic psychedelic use and three markers of physical health (self-reported overall health, body mass index, and heart condition and/or cancer in the past 12 months) while controlling for a range of covariates.

**Results:**

Respondents who reported having tried a classic psychedelic at least once in their lifetime had significantly higher odds of greater self-reported overall health and significantly lower odds of being overweight or obese versus having a normal weight. The association between lifetime classic psychedelic use and having a heart condition and/or cancer in the past 12 months approached conventional levels of significance, with lower odds of having a heart condition and/or cancer in the past 12 months for respondents who had tried a classic psychedelic at least once.

**Conclusion:**

The results of the present study suggest that classic psychedelics may be beneficial to physical health. Future research should investigate the causal effects of classic psychedelics on physical health and evaluate possible mechanisms.

## Introduction

The effects of psychedelic drug use on human cognition and behaviour have recently received significant scientific attention (Rucker et al., 2018; Sessa, 2018). The research has been conducted in controlled settings and has primarily focused on classic psychedelics, which are a subclass of psychedelics, with little evidence of physiological toxicity, known to act as agonists primarily at 5-HT2A receptors (dos Santos et al., 2018; Johnson et al., 2018; Winkelman, 2014). The three main classes of classic psychedelics (tryptamines, lysergamides and phenethylamines) are distinguished by unique chemical structures and neurochemical mechanisms (Szabo, 2015). Most notably, they include N,N-dimethyltryptamine (DMT), the DMT-containing admixture ayahuasca, psilocybin, lysergic acid diethylamide (LSD), mescaline, and the mescaline-containing cacti peyote and San Pedro (Sexton et al., 2019a).

The evidence to date suggests that classic psychedelics have immunomodulatory and anti-inflammatory properties (Flanagan and Nichols, 2018; Frecska et al., 2016; Nichols, 2009; Szabo, 2015, 2019); carry low risk of adverse effects when administered by health professionals in a safe and supportive environment (Nutt and Carhart-Harris, 2020; Nutt et al., 2010; Rucker et al., 2018); and can be effective in the treatment of depression, anxiety and addiction (Aday et al., 2020; Carhart-Harris et al., 2016, 2018; Davis et al., 2020; Goldberg et al., 2020; Johnson et al., 2014; Krebs and Johansen, 2012; Luoma et al., 2020). For example, patients with treatment-resistant depression experienced reductions in depressive symptoms after two oral doses of psilocybin. There was no control group in the study, but the depressive symptoms remained significantly reduced at 1 week, 3 months, and 6 months post-treatment (Carhart-Harris et al., 2016, 2018). The patients were interviewed post-treatment and many of them reported significant improvements in health behaviour (Watts et al., 2017), which suggests that classic psychedelic use might induce behavioural changes favourable to physical health.

While double-blind, randomized, placebo-controlled trials are warranted to experimentally examine the effects of classic psychedelics on physical health, population studies can provide insight into these knowledge gaps. The National Survey on Drug Use and Health (NSDUH) has frequently been analyzed to provide weighted estimates of the prevalence and associations of lifetime classic psychedelic use in the United States. The findings have varied from 13.4 to 13.6% of the adult population reporting lifetime classic psychedelic use (Hendricks et al., 2017; Johansen and Krebs, 2015) and have consistently showed that lifetime classic psychedelic users are more likely to be male, white, younger than 65 years of age, and have higher income and education (Johansen and Krebs, 2015; Krebs and Johansen, 2013a, 2013b). The results have also shown that lifetime classic psychedelic use is associated with lower odds of psychological distress and suicidality (Hendricks et al., 2015), lower odds of opioid abuse and dependence (Pisano et al., 2017), and lower odds of criminal behavior (Hendricks et al., 2017), which broadly mirrors the research that suggests therapeutic efficacy of classic psychedelics as well as the low risk of harm to self and others that classic psychedelics have been ascribed by drug experts in the United Kingdom (Nutt et al., 2010), the Netherlands (Van Amsterdam et al., 2010) and Australia (Bonomo et al., 2019). There have, in other words, been several population studies on lifetime classic psychedelic use, but the association between lifetime classic psychedelic use and physical health remains unexplored.

Using pooled data from the NSDUH (2015–2018), the present study seeks to investigate the association between lifetime classic psychedelic use and three markers of physical health (self-reported overall health, body mass index (BMI), and heart condition and/or cancer in past 12 months). We hypothesized that lifetime classic psychedelic use would be associated with better physical health status.

## Materials and methods

### Data and population

The NSDUH is an annual survey designed to measure the prevalence of substance use and mental health issues in the United States. The present study used pooled data from the NSDUH survey years 2015 to 2018, which were weighted to reflect the civilian noninstitutionalized population and contained responses from 171,766 (unweighted) adults aged 18 or above. The NSDUH sampling and questionnaire methodology are described on their website: https://nsduhweb.rti.org/respweb/about_nsduh.html

### Variables

The dependent variables were self-reported overall health (variable HEALTH2 recoded; 1 = Fair/Poor, 2 = Good, 3 = Very Good, 4 = Excellent), BMI (variable BMI2 recoded per National Institute of Health (NIH) guidelines (National Heart, Lung, and Blood Institute (NHLBI) 1998): 1 = Underweight (<18.5), 2 = Normal Weight (18.5–25), 3 = Overweight (25–30), 4 = Obesity – Class 1 (30–35), 5 = Obesity – Class 2 (35–40), 6 = Extreme Obesity – Class 3 (>40)), and heart condition and/or cancer in past 12 months (variables HRTCONDYR and CANCERYR combined such that a ‘yes’ response to either variable was coded as 1 = Yes whereas a ‘no’ response to both variables was coded as 0 = No).

The independent variables were DMT (code 616 from variables HALLUCOT1, HALLUCOT2, HALLUCOT3, HALLUCOT4 and HALLUCOT5), ayahuasca (an entheogenic brew that contains DMT; code 6103 from variables HALLUCOT1, HALLUCOT2, HALLUCOT3, HALLUCOT4 and HALLUCOT5), psilocybin (variable PSILCY2 = 1), LSD (variable lsdflag = 1), mescaline (variable MESC2 = 1), and peyote or San Pedro (cacti that contain mescaline; variable PEYOTE2 = 1 or code 6077 from variables HALLUCOT1, HALLUCOT2, HALLUCOT3, HALLUCOT4 and HALLUCOT5). Respondents reporting that they had ever, even once, used any of the above classic psychedelics were coded as positive for lifetime classic psychedelic use, whereas those indicating that they had never used any of these substances were coded as negative. The question in the NSDUH concerning use of DMT, alpha-methyltryptamine (AMT) and 5-methoxy-N, N-diisopropyltryptamine (5-MeO-DIPT) (variable DAMTFXFLAG: ‘Have you ever, even once, used any of the following: DMT, also called dimethyltryptamine, AMT, also called alpha-methyltryptamine, or Foxy, also called 5-MeO-DIPT?’) was not included, because neither AMT nor 5-MeO-DIPT are classified as classic psychedelic and DMT use alone could not be determined from the question.

The control variables were age in years (variable CATAG6; 18–25, 26–34, 35–49, 50–64 or 65 or older); sex (variable IRSEX; male or female); sexual orientation (variable SEXIDENT; heterosexual, lesbian or gay, bisexual); ethnoracial identity (variable NEWRACE2; non-Hispanic White, non-Hispanic African American, non-Hispanic Native American/Alaska Native, non-Hispanic Native Hawaiian/Pacific Islander, non-Hispanic Asian, non-Hispanic more than one race or Hispanic); educational attainment (variable IREDUHIGHST2; 5th grade or less, 6th grade, 7th grade, 8th grade, 9th grade, 10th grade, 11th or 12th grade completed, High school diploma/GED, some college credit but no degree, Associate’s degree, College degree or higher), annual household income (variable INCOME; less than US$20,000, US$20,000–49,999, US$50,000–74,999 or US$75,000 or more); marital status (variables IRMARITSTAT and IRMARIT recoded; married, divorced/separated, widowed or never married); self-reported engagement in risky behaviour (variable RSKYFQTES recoded; never, seldom, sometimes or always), lifetime cocaine use (variable COCFLAG; ever used or never used), lifetime other stimulant use (variable STMANYFLAG; ever used or never used), lifetime sedative use (variable SEDANYFLAG; ever used or never used), lifetime tranquilizer use (variable TRQANYFLAG; ever used or never used), lifetime heroin use (variable HERFLAG; ever used or never used), lifetime pain reliever use (variable PNRANYFLAG; ever used or never used), lifetime marijuana use (variable MRJFLAG; ever used or never used), lifetime phencyclidine (PCP) use (variable PCPFLAG; ever used or never used), lifetime 3,4-methylenedioxymethamphetamine (MDMA/ecstasy) use (variable ECSTMOFLAG; ever used or never used), lifetime inhalant use (variable INHALFLAG; ever used or never used), lifetime smokeless tobacco use (variable SMKLSSFLAG; ever used or never used), lifetime pipe tobacco use (variable PIPFLAG; ever used or never used), lifetime cigar use (variable CGRFLAG; ever used or never used), lifetime daily cigarette use (variable CDUFLAG; ever used or never used) and age of first alcohol use (variable IRALCAGE recoded; less than 13 years of age (Preteen), 13–19 years of age (Teen), more than 19 years of age (Adult), or never used). These control variables were coded as separate covariates and broadly mirror the covariates of prior investigations (Hendricks et al., 2015; Sexton et al., 2019b), except for lifetime smokeless tobacco, pipe tobacco, cigar and daily cigarette use, as well as age of first alcohol use, which were added to control for a lifetime history of major health risk factors (Christensen et al., 2018; Hu et al., 2017; Inoue-Choi et al., 2019a, 2019b; Levola et al., 2020). Lastly, a recoded version of the Kessler Psychological Distress Scale (Kessler et al. 2002, 2010) was also included as a control variable (variable K6SCMON recoded into dichotomous variable), but only in the ordered logistic regression model predicting self-reported overall health (see below) to ensure that self-reported overall health was not influenced by the mental health status of the respondents.

### Statistical analyses

The present study used descriptive statistics to present an overview of lifetime use of classic psychedelics in the United States (Table 1). Multiple regressions were used to calculate adjusted odds ratios with 95% confidence intervals and examine the unique associations between lifetime classic psychedelic use and markers of physical health. Ordered logistic regression was used to examine the association between lifetime classic psychedelic use and self-reported overall health (Table 2); multinomial logistic regression was used to examine the association between lifetime classic psychedelic use and BMI (Table 3); and logistic regression was used to examine the association between lifetime classic psychedelic use and having a heart condition and/or cancer in the past 12 months (Table 4).

**Table 1.** Lifetime classic psychedelic (DMT, Ayahuasca, Psilocybin, LSD, Mescaline, Peyote or San Pedro) users in the United States (2015–2018).

Responses	% (95% CI)	Population estimates
Ever used	13.8 (13.5–14.1)	33,925,666
Never used	86.2 (85.9–86.5)	211,912,497
Total	100	245,838,163

Note: The number of observations was 171,766 (unweighted). The percentages have been weighted to reflect national estimates and have been rounded to the closest decimal point.

**Table 2.** Lifetime classic psychedelic use and self-reported overall health.

	aOR (95% CI)	*p* value	*N*
Self-reported overall health	1.08 (1.02–1.14)	.0048	168,123

aOR: adjusted odds ratio; CI: confidence interval; *N* refers to unweighted counts in the regression model; odds ratios are adjusted for age, sex, sexual orientation, ethnoracial identity, educational attainment, annual household income, marital status, self-reported engagement in risky behaviour, lifetime use of cocaine, other stimulants, sedatives, tranquilizers, heroin, pain relievers, marijuana, phencyclidine (PCP), 3,4-methylenedioxymethamphetamine (MDMA/ecstasy), inhalants, smokeless tobacco, pipe tobacco, cigar and cigarettes daily, age of first alcohol use, and past month psychological distress.

**Table 3.** Lifetime classic psychedelic use and body mass index.

	aRRR (95% CI)	*p* value	*N*
Normal weight (Reference)			56,955
Underweight	0.93 (0.72–1.20)	. 5753	3940
Overweight	0.86 (0.80–0.93)	.0002	51,212
Obesity – Class 1	0.80 (0.74–0.87)	<.0001	28,913
Obesity – Class 2	0.76 (0.69–0.83)	<.0001	13,831
Extreme obesity – Class 3	0.78 (0.68–0.88)	.0002	8926

aRRR: adjusted relative risk ratio; CI: confidence interval; *N* refers to unweighted counts in each row; relative risk ratios are adjusted for age, sex, sexual orientation, ethnoracial identity, educational attainment, annual household income, marital status, self-reported engagement in risky behaviour, lifetime use of cocaine, other stimulants, sedatives, tranquilizers, heroin, pain relievers, marijuana, phencyclidine (PCP), 3,4-methylenedioxymethamphetamine (MDMA/ecstasy), inhalants, smokeless tobacco, pipe tobacco, cigar and cigarettes daily, and age of first alcohol use.

**Table 4.** Lifetime classic psychedelic use and heart condition and/or cancer in the past year.

	aOR (95% CI)	*p* value	*N*
Heart condition and/or cancer in the past year	0.89 (0.77–1.02)	.0917	168,147

aOR: adjusted odds ratio; CI: confidence interval; *N* refers to unweighted counts in the regression model; odds ratios are adjusted for age, sex, sexual orientation, ethnoracial identity, educational attainment, annual household income, marital status, self-reported engagement in risky behaviour, lifetime use of cocaine, other stimulants, sedatives, tranquilizers, heroin, pain relievers, marijuana, phencyclidine (PCP), 3,4-methylenedioxymethamphetamine (MDMA/ecstasy), inhalants, smokeless tobacco, pipe tobacco, cigar and cigarettes daily, and age of first alcohol use. Note: the odds ratios are similar when heart condition in the past year and cancer in the past year are analyzed as separate dependent variables (see Tables A1 to A3 in Appendix for more information).

The analyses used weights provided by the NSDUH, and the control variables listed above were included as covariates in the regression models to control for potential sources of confounding. Insofar that the NSDUH is a nationally representative survey, there was no a priori rationale for identifying or removing outliers. The NSDUH conducts statistical imputation and revision for missing values for select variables, denoted by ‘IMP’ or ‘IR’ prefixes. A number of constraints are put in place by the NSDUH to ensure consistency in imputed values with non-missing values for use in multivariate analyses. In the present analyses we did not conduct additional imputations beyond what the NSDUH has already provided in their annual releases. All missing values were treated as missing. Additional information on the NSDUH imputation procedure can be found in the ‘Statistical Imputation’ section in the introduction of each annual codebook. Finally, though there was no control for multiple comparisons, exact p-values are reported to the fourth decimal place, which allows for the application of conservative Bonferroni-type corrections of the reader’s choosing (SAMHSA, 2019).

## Results

### Frequency distributions

Table 1 presents descriptive statistics of lifetime classic psychedelic users in the United States (2015–2018). As shown in the table, approximately 14% of the sample reported lifetime classic psychedelic use, which suggests that almost 34 million American adults have used a classic psychedelic at least once in their lifetime, based on the population estimates from the NSDUH.

### Multiple regressions

Table 2 presents results from the ordered logistic regression on the association between lifetime classic psychedelic use and self-reported overall health. As illustrated in the table, lifetime classic psychedelic use was associated with significantly higher odds of greater self-reported overall health.

Table 3 presents results from the multinomial logistic regression on the association between lifetime classic psychedelic use and BMI. As illustrated in the table, lifetime classic psychedelic use was associated with significantly lower odds of being overweight or obese as compared to having a normal weight.

Table 4 presents results from the logistic regression on the association between lifetime classic psychedelic use and having a heart condition and/or cancer in the past year. As illustrated in the table, the association between lifetime classic psychedelic use and having a heart condition and/or cancer in the past 12 months approached conventional levels of significance, with lower odds of having a heart condition and/or cancer in the past 12 months for respondents who had tried a classic psychedelic at least once.

## Discussion

The present study investigated the association between lifetime classic psychedelic use and three markers of physical health (self-reported overall health, BMI, and heart condition and/or cancer in the past 12 months). Findings show that respondents who reported having ever used a classic psychedelic had significantly higher odds of greater self-reported overall health and significantly lower odds of being overweight or obese as compared to having a normal weight. The association between lifetime classic psychedelic use and having a heart condition and/or cancer in the past 12 months approached conventional levels of significance, with lower odds of having a heart condition and/or cancer in the past 12 months for respondents who had tried a classic psychedelic at least once. Taken together, these results suggest that classic psychedelics may have long-term beneficial effects beyond improved mental health.

While the acute transcendent experience occasioned by classic psychedelics may presumably induce long-term changes in health behaviour that contribute to better physical health, it is plausible that there are other key mechanisms through which classic psychedelics could influence physical health, including improvements on various indices of mental health beyond the simple absence of psychological distress (e.g. increased prosociality, trait mindfulness and purpose in life; Griffiths et al., 2018; Murphy-Beiner and Soar, 2020), many of which are well-known risk factors for physical maladies (Chaddha et al., 2016; Germann, 2020; Hernandez et al., 2018); immunomodulatory and anti-inflammatory effects of relevance to physical health (Flanagan and Nichols, 2018; Frecska et al., 2013, 2016; Szabo, 2015, 2019; Szabo et al., 2014; Thompson and Szabo, 2020; Tourino et al., 2013; Winkelman and Sessa, 2019); and high affinity to receptor subtypes (e.g. serotonin 2A receptors) that are implicated in the pathophysiology of different physical disorders (Nichols, 2009; Thompson and Szabo, 2020). Future research is needed to better understand potential causal pathways of classic psychedelics on physical health.

There are several limitations with the present study that need serious consideration before the results are interpreted. First, the cross-sectional design of the study limits causal inference. The analyses controlled for multiple sources of potential confounding, but the associations might have been obscured by response bias or latent variables that were not controlled for (e.g. a common factor predisposing one to classic psychedelic use may also predispose one to healthy lifestyle behaviours including physical activity). Second, the dataset did not contain information on frequency of classic psychedelic use, dose used or context of use. The present study could therefore not evaluate frequency, dose or context-specific relationships between classic psychedelic use and physical health markers. Third, it is also not possible to rule out that classic psychedelic use might have caused harm on the individual level, even if it did not obfuscate the population-level associations. Fourth, given the potential importance of immunomodulatory and inflammatory factors in the current study, it would have been sensible to also control for regular anti-inflammatory drug (e.g. nonsteroidal anti-inflammatory drug (NSAID)) use, but assessment of this behaviour was not included in the NSDUH. Fifth, BMI has been widely used as a screening tool for overweight or obesity, but it does not account for details such as fat distribution, which limits its utility as a marker of physical health (Prentice and Jebb, 2001). Finally, it is noted that some associations of lifetime classic psychedelic use were somewhat modest in size (e.g. heart condition and/or cancer in the past year). However, even modest effects can have substantial impacts at the population level. For instance, considering approximately 1.2 million people die from heart disease or cancer every year in the United States alone (Heron, 2019), even a small decrease (e.g. 11%) in the prevalence of these illnesses could translate to thousands of lives saved annually.

## Conclusion

The psychedelic research to date has primarily focused on mental health, but relatively little is known about how classic psychedelics might influence physical health. The findings in the present study suggest that lifetime classic psychedelic use is associated with higher odds of better physical health status, which demonstrates the need for more rigorous research to better understand potential causal pathways of classic psychedelics on physical functioning.

## Appendix

### Logistic regressions

**Table A1.** Lifetime classic psychedelic use predicting heart condition in the past year.

	aOR (95% CI)	*p* value	*N*
Heart condition in the past year	0.90 (0.76–1.06)	.2074	168,147

aOR: adjusted odds ratio; CI: confidence interval; *N* refers to unweighted counts in each regression model; odds ratios are adjusted for age, sex, sexual orientation, ethnoracial identity, educational attainment, annual household income, marital status, self-reported engagement in risky behaviour, lifetime use of cocaine, other stimulants, sedatives, tranquilizers, heroin, pain relievers, marijuana, phencyclidine (PCP), 3,4-methylenedioxymethamphetamine (MDMA/ecstasy), inhalants, smokeless tobacco, pipe tobacco, cigar and cigarettes daily, and age of first alcohol use.

**Table A2.** Lifetime classic psychedelic use predicting cancer in the past year.

	aOR (95% CI)	*p* value	*N*
Cancer in the past year	0.88 (0.68–1.14)	.3329	168,147

aOR: adjusted odds ratio; CI: confidence interval; *N* refers to unweighted counts in each regression model; odds ratios are adjusted for age, sex, sexual orientation, ethnoracial identity, educational attainment, annual household income, marital status, self-reported engagement in risky behaviour, lifetime use of cocaine, other stimulants, sedatives, tranquilizers, heroin, pain relievers, marijuana, phencyclidine (PCP), 3,4-methylenedioxymethamphetamine (MDMA/ecstasy), inhalants, smokeless tobacco, pipe tobacco, cigar and cigarettes daily, and age of first alcohol use.

### Multinomial logistic regression

**Table A3.** Lifetime classic psychedelic use predicting heart condition only, cancer only, and both heart condition and cancer in the past year.

	aRRR (95% CI)	*p* value	*N*
No heart condition or cancer (Reference)			161,036
Heart condition only in the past year	0.89 (0.76–1.05)	. 1752	5563
Cancer only in the past year	0.86 (0.67–1.11)	.2452	1328
Both heart condition and cancer in the past year	0.98 (0.43–2.25)	.9681	220

aRRR: adjusted relative risk ratio; CI: confidence interval; *N* refers to unweighted counts in each row; relative risk ratios are adjusted for age, sex, sexual orientation, ethnoracial identity, educational attainment, annual household income, marital status, self-reported engagement in risky behaviour, lifetime use of cocaine, other stimulants, sedatives, tranquilizers, heroin, pain relievers, marijuana, phencyclidine (PCP), 3,4-methylenedioxymethamphetamine (MDMA/ecstasy), inhalants, smokeless tobacco, pipe tobacco, cigar and cigarettes daily, and age of first alcohol use.
